# Subthreshold Laser Ablation Measurements by Langmuir Probe Method for ns Irradiation of HfO_2_ and ZrO_2_

**DOI:** 10.3390/ma16020536

**Published:** 2023-01-05

**Authors:** Radu Udrea, Stefan Andrei Irimiciuc, Valentin Craciun

**Affiliations:** 1 Apel Laser, Street Vanatorilor 25, Mogosoaia, 077135 Ilfov, Romania; 2 Physics Faculty, University of Bucharest, 077125 Magurele, Romania; 3 National Insitute for Lasers Plasma and Radiation Physics, 077125 Magurele, Romania; 4 Extreme Light Infrastructure for Nuclear Physics, 077125 Magurele, Romania

**Keywords:** ablation threshold, ablation mechanisms, subthreshold measurement, Langmuir probe, ion acceleration

## Abstract

The unbiased Langmuir probe (LP) method was used to perform measurements on HfO_2_ and ZrO_2_ samples around the laser ablation threshold on a wide range of irradiation conditions. Important changes in the lifetime (from ms to μs) and the shape of the charge particle current were seen with the increase of the laser fluence. The ablation threshold was estimated by evaluating the overall average ablated charge as a function of the laser fluence. Above the ablation threshold, the generation of high kinetic species is seen, which can reach several keV. An important jump in ion acceleration potential is observed for values above 1 J/cm^2^, which coincides with the dominant presence of negative ions in the plasma. The evolution of several plasma parameters (ion density, expansion velocity, electron temperature, Debye length) was investigated and correlated with the fundamental ablation mechanism involved in various irradiation regimes. The LP data were correlated with COMSOL simulations on the maximum surface temperature reached during irradiation. Important correlations between the evaporation and melting processes and ablation threshold fluence and ion acceleration phenomena are also reported.

## 1. Introduction

Recent developments in high power laser sources have led to the accelerated development of two important research areas, one focused on the fundamentals of short and ultrashort laser–matter interaction, and the second one concerning the manufacturing of suitable materials (optical components) with prolonged lifetime in the laser beam lines. This approach has raised an important question regarding real-time monitoring tools, which can provide accurate feedback for fluences below the laser damage threshold and the complete destruction of the material. HfO_2_ and ZrO_2_ in thin film form have been promising coating materials for mirrors for high power fs laser systems. Both HfO_2_ and ZrO_2_ have been intensively studied due to their low absorption coefficients, high transmittance, high refractive index, and high laser damage threshold, as well as the possibility to be combined with silicon dioxide to form multi-layers [1,2,3]. Previously, reported values for their laser ablation threshold show a significant variation. In the case of HfO_2_ values, they have been reported anywhere from 1 to 20 J/cm^2^. The lower values generally correspond to a UV-irradiation regime, a shorter pulse duration and, in the case of thin films, lower thicknesses [4,5,6]. In the case of ZrO_2_, similar maxima values were obtained for thin films, primarily for 1064 nm laser irradiation with remarkable effects reported when decreasing the thickness to a monolayer [7]. Moreover, small improvements in the increase of laser-induced damage threshold (LIDT) values have been achieved through high-temperature annealing [8] and laser conditioning [7]. For both materials it was shown that repeated irradiation provoked a laser-induced fatigue behavior, decreasing the LIDT over many pulses [9]. Various techniques have been used in the literature for the determination of the laser ablation threshold. Among these several can be named: optical microscopy [5], profilometry [10,11], plasma emission by photodiode [12], camera damage test (CCD, CMOS) [13], interferometric microscopy [14], plasma emission spectrometry [15], and Langmuir probe plasma diagnostics [16,17]. For ultrashort pulsed lasers, the ablation threshold has also been computed by implementing complex theoretical models [16]. There are various drawbacks for each of these techniques, ranging from surface measurement error, necessity to carry out the measurements ex situ, time requirements, costs, and difficulty of implementation. Alternatively, one can look towards the existing diagnostic tools for monitoring the laser ablated material. The Langmuir probe (LP) is a proven technique suitable for charge detection on a wide temporal scale (ps–ms), which covers the complete formation and expansion of the resulting laser-produced plasma (LPP) generated above the ablation threshold as it detects contributions from each stage [18]. Thus, the implementation of LP with complementary target current (TC), optical emission spectroscopy, or mass spectrometry measurements can provide the development of robust in situ and real time diagnostics. Understanding the behavior of a specific material around the laser damage threshold has relevance for several applications, such as laser patterning, cleaning, pulsed laser deposition, laser annealing process, or laser-induced breakdown spectroscopy laser just to name a few. An electrical diagnostic system can provide information over the working status of the optical components during ns or fs laser experiments and offer early warning for the appearance of incubation effects induced by multi-pulses irradiation. Although published reports about fs-incubation stage detection using LP cannot be found, there are some reports on the use of LP technique for ablation threshold estimation in the ns regime [19]. The majority of the data available in the literature regarding LP measurements concerns the dynamic of the charged particle above the ablation threshold, mostly at large distances (over 2–3 cm) from the target [20] and at various measurement angles with respect to the main expansion axis (defined as the axis orthogonal on the irradiation plane of the target). However, the key to prolonging the life of the optical components is to understand and characterize the electrical signals collected by LP below the ablation threshold. The concept of the ablation threshold is strongly related to the fundamental mechanisms of ns or fs laser–matter interactions, which have been investigated in an impressive number of papers and usually follow a complex trail of electrostatic and thermal interactions with one common underline: the first stages of laser ablation are of an electrostatic nature, while the secondary stages are of a thermal nature. A complete description of the ablation process would require simultaneous analysis of all the mechanisms and the existing coupling between them. Briefly, the process begins with the single- or multi-photon absorption and subsequent material excitation, and for longer pulse duration, the result is often a rise in temperature, which can induce material evaporation or explosive-type ablation (explosive boiling). If the photon energy is high enough it will result in direct bond breaking leading to desorption of single atoms, ions, electrons, molecules, clusters, or surface fragmentation. All of these processes are defined by specific temporal ranges, which are related to the bond strength and overall thermal and optical properties of the irradiated material.

In this paper, we report on subthreshold measurements of ns laser beam irradiation of HfO_2_ and ZrO_2_ samples performed by the unbiased LP technique. Changes in particle distribution and ion kinetic energy are found as markers of the threshold fluence. The effect of laser fluence on several plasma properties is investigated with a key interest in ionic acceleration potential. Velocity and acceleration fields are investigated as a function of the laser fluence as well as changes in densities of both oxygen and metallic species.

## 2. Materials and Methods

**Figure 1:**
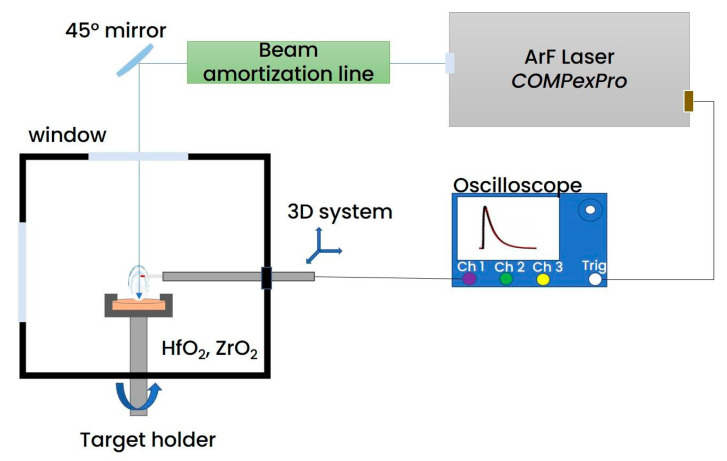
Experimental set-up for the LP measurements.

An ArF excimer laser (λ = 193 nm, pulse duration τ = 25 ns,) was used to irradiate cylindrical (φ= 1.2 cm × 0.5 cm) HfO_2_ and ZrO_2_ targets (Neyco, France) at various fluences (0.6–5.0 J/cm^2^) placed in a reaction chamber vacuumed down to 2 × 10^−4^ mbar. The experiments were performed in single shot mode with the samples being rotated so each irradiation is performed on pristine surfaces, with the samples being polished before each experimental series. The targets were electrically grounded during the experiments. A 2 mm long, 0.5 mm diameter tungsten wire was used as an active region of a Langmuir probe (LP) placed at 1 cm away from the target, parallel to the target surface. The LP was placed off axis with respect to the irradiation plane to avoid shadowing effects or potential interactions between the incoming beam and the LP (see Figure 1).

## 3. Results and Discussion

**Figure 2:**
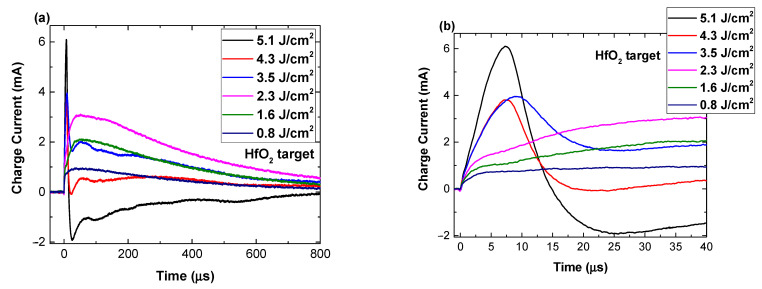
Unbiased LP current temporal traces recorded at 1 cm from the target for different irradiation fluence conditions presented for long (**a**) and short time scales (**b**).

In Figure 2, the temporal traces of the charge current recorded by the LP in various irradiation conditions have been represented. Clear changes in both structure and distribution of the current across the lifetime of the plasma are visible. For low irradiation values (0.8 J/cm^2^), a single peak distribution with maximum defining particles traveling with 290 m/s and a low current density was observed. The increase in the laser irradiation conditions reveals a split of the current (first seen at 1.6 J/cm^2^) in two peaks defining Hf ions traveling with 2.5 km/s and 208 m/s. The split of the current trace is correlated with the different mechanism involved in the ablation process and by the differential acceleration of multiple ionized ions occurring at the front of the plasma by the transient double layer. The increase of laser fluence leads to an increase in acceleration potential, and thus, charged particle kinetic energy. Other studies have shown through optical methods that velocities in the ranges found here correspond to atomic and clusters or heavier particles [21,22]; however, in this study, we only use LP, which is tailored only for the measurement of charged particles. The differences can be induced by the properties of the laser (wavelength, duration, peak fluence, etc.) [23,24,25,26]. With the further increase of the fluence, several new maxima were seen. This phenomenon acts like a separation induced by the mass-charge/ratio, which could only be induced by the presence of an accelerating field in the incipient moment of the ablation. Thus, it can be concluded that the presence of multiple maxima is a clear signature of the Coulomb explosion mechanism [27,28,29]. For fluences above 4 J/cm^2^, observed negative peaks are also observable, which correspond to the oxygen contribution to the current. This phenomenon has a clear energetic threshold; there is a factor of 2 in ionization energy of O (13.6 eV) when compared to Hf (6.8 eV). The results are in good agreement with our previous reports on CuO [30] and CuI [31] plasmas, where a multiple structure of the ionic current was explained as a result of multiple ionization states of the metallic species in the plasma.

**Figure 3:**
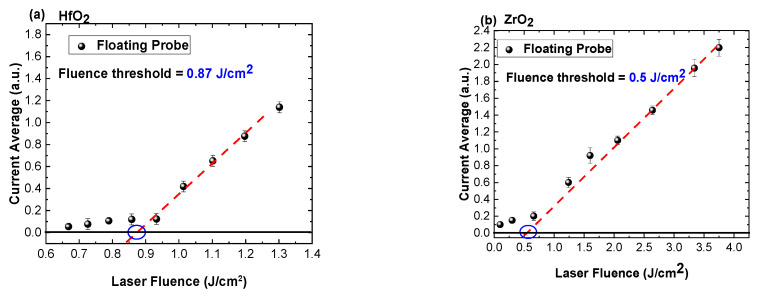
Current average representation as function of laser fluence for HfO_2_ (**a**) and ZrO_2_ (**b**).

By extending the range of fluences for which the unbiased current from the laser evaporated cloud can be collected, some steps can be taken towards determination of the ablation threshold fluence. Previous reports have used the saturation current, defined as the temporal trace characteristics, for a bias, which will describe the saturation ionic region in the I–V characteristic. In [29], it was shown that for the ablation of ceramic targets the saturation currents are offering an incomplete information from the plasma by convoluting the contribution from each energetic species presented in the plasma and limiting the contribution from high energetic species. According to [24], by representing the total charge, defined as the current average over the lifetime of the plasma, as a function of the laser fluence, one can determine the threshold fluence as the inflection point of the function. Similar functions for ablation threshold determination have been proposed [5,15,32] by using the ablation crater area or ablation depth. The values obtained, 0.87 J/cm^2^ for HfO_2_ and 0.5 J/cm^2^ for ZrO_2_, as seen in Figure 3, are slightly below other reports from the literature [5,33,34]. This is due to the sensitivity of the technique, especially considering the exponential increase in charge current and the appearance of high kinetic ionic peaks and the lower quality of the surface morphology.

**Figure 4:**
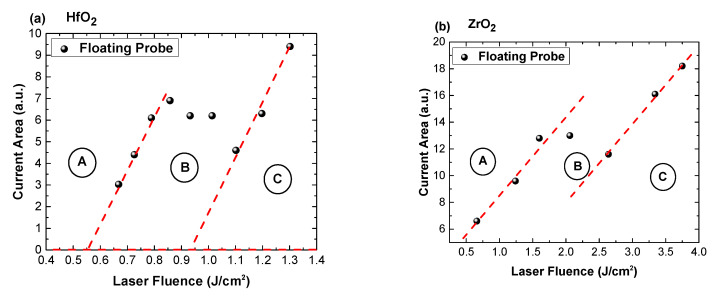
Total area of the LP current as a function of laser fluence for HfO_2_ (**a**) and ZrO_2_ (**b**).

As previous reports using the LP have only considered the saturation current, an alternative representation will be considered in the following. The shape of the ionic temporal traces, as it can be seen in Figure 2, has multiple contributions from Hf ions and O ones depending on the irradiation conditions. By representing the total current area (including possible contributions from O^-^ charges) as a function of the laser fluence (Figure 4a,b), further insight into the transition from a single energetic peak to the rather complex multi-peak structure seen at high fluences was obtained. Both figures depict similar trends with linear behavior in the 0.6–0.87 J/cm^2^ range (region A), followed by a plateau in the 0.87–1.0 J/cm^2^ (region B), and another linear evolution for values higher than 1 J/cm^2^ (region C). It is notable that both linear regions are defined by the same slope (2 ×10^−4^ for HfO_2_ and 6.4 for ZrO_2_). Similar evolution is seen for ZrO_2_; however, the values are shifted above the threshold value.

**Figure 5:**
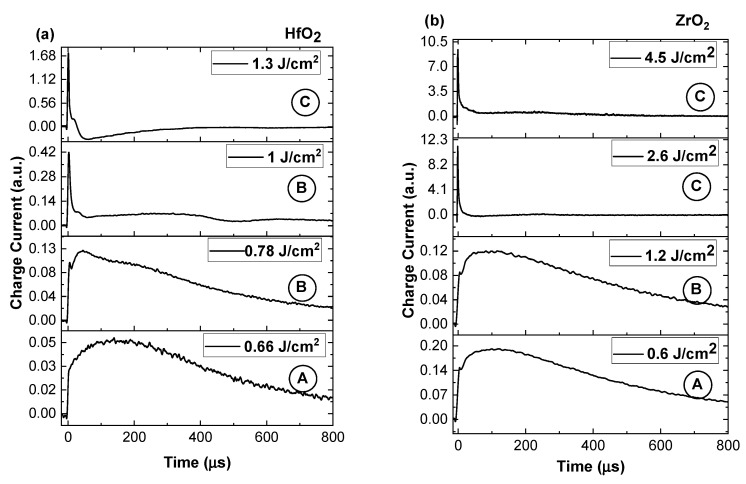
Time of arrival charge traces recorded for HfO_2_ (**a**) and ZrO_2_ (**b**) characterizing the three fluence regimes (the A, B, C, corresponds to the regimes identified in Figure 4).

Further investigations concern a comparative representation of charge currents characterizing each specific area (Figure 5a for HfO_2_ and Figure 5b for ZrO_2_). This representation highlights the transition discussed in Figure 2 and adds context in terms of the relation between the irradiation conditions and the ablation threshold. It can be clearly seen that for fluences below the threshold value found here, the charge currents are characterized by a long lifetime (around 800 μs for both materials). In these irradiation conditions it is difficult to assume any acceleration of the ions. The best scenario that would describe the observed behavior is the laser cleaning effect (i.e., removal of weakly bounded particles and adsorbed molecules). This process precedes any thermal mechanism and it can be described by low electrostatic interactions. Around the threshold value, the current temporal trace presents multiple maxima, which suggests the presence of multiple ionized species reaching the probe at different moments in time. This separation implies the presence of an accelerating field, which induces the separation based on a charge-to-mass ratio value. Around the threshold value, the gentle ablation regime was identified, as defined by N. Bulgakova’s group in [28], as being defined by long lifetime with contributions from various ionized states. A significant change is seen through the appearance of a highly energetic group of ions and increase of about one order of magnitude in the overall charge density. Above the threshold, the complete temporal range of the charge currents significantly changes with a positive ionic contribution seen in the tens of μs regime. This is consistent with other reports for laser produced plasma [35]. It is worth noting that in all investigated conditions a negative contribution was observed, which is characterized by a short arrival time and a consistent density of one order of magnitude lower than the main ionic peak. The presence of this electron peak confirmed the electrostatic mechanism as the dominant one in material removal, with the expected thermal effects (melting and explosive boiling) only being seen at the affected surface.

**Figure 6:**
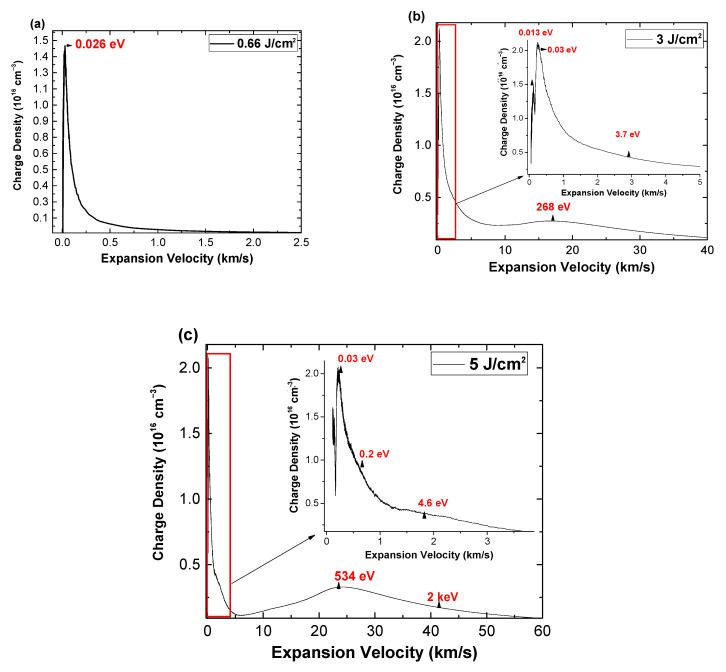
HfO_2_ plasma charge density distribution with expansion velocity characterizing different irradiation conditions (0.66 J/cm^2^ (**a**); 3 J/cm^2^ (**b**) and 5 J/cm^2^ (**c**)).

In order to explore the kinetic regimes of the plasmas generated in various irradiation regimes, the approach from [18] was implemented. The charge density distribution with the expansion velocity were derived and plotted in Figure 6. For the study performed on the HfO_2_ sample, some strong changes can be seen for the two regimes. When the laser fluence is below the threshold value (Figure 6a), the velocity distribution extends up to 1.5 km/s, with the core of the plasma having 0–0.5 km/s, with the most probable kinetic energy of the ablated particle cloud being 2.6 × 10^−2^ eV. Indeed, the distribution is characteristic for the expansion of particles in a low kinetic regime. Above the ablation regime for fluence values relevant to the pulsed laser deposition conditions (Figure 6b), a splitting of the distribution into multiple peaks is observed. The slow peak into 1.3 × 10^−2^ eV and 3 × 10^−2^ eV with an additional 3.7 eV tail, while the presence of a new peak characterizing a high kinetic expansion regime with a most probable energy of 268 eV. A further increase in laser fluence lead to the further shift of the slow peak into a 0.2 eV group and an increase of 0.9 eV of the secondary peak energy. The high kinetic group of ions have their most probable kinetic energy shifted up to 584 eV with the tail reaching 2 keV. These values are highly relevant for both high-power laser facilities where the kinetic energy will define a traveling distance of the ablated debris [33] and for pulsed laser deposition where the kinetic of the particle in the plasma phase can induce different growth mechanisms, defects, or special morphological features [33]. For the ZrO_2_ sample, the distribution followed a similar evolution, with overall higher values of the kinetic energy by a factor of 3.6.

**Figure 7:**
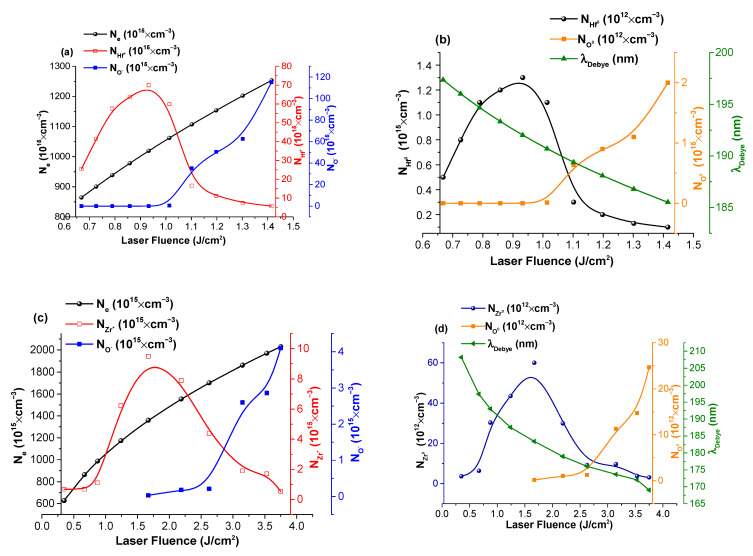
Particle densities and Debye length evolution with laser fluence for HfO_2_ (**a**,**b**) and ZrO_2_ (**c**,**d**).

A more in-depth analysis of charge current temporal trace can be done by following the approaches previously used by our group in [34,35] and by Torrisi in [36,37,38], by knowing the irradiation conditions and a series of basic plasma parameters information on the electron and atomic density, as well as the Debye length. When representing these parameters as a function of the laser fluence (Figure 7a–d), some important dependences can be seen. The increase in laser fluence leads to an expected increase in electron density from 0.85 × 10^18^ cm^−3^ to 1.25 × 10^18^ cm^−3^ for HfO_2_, and from 0.6 × 10^18^ to 2× 10^18^ cm^−3^ for the ZrO_2_, with the Debye length decreasing in the 0.20–0.18 μm range. The representation of the Hf charge density as a function of laser fluence sees an increase of a factor of 3 up to a maximum of 7.2 × 10^15^ cm^−3^ at 0.9 J/cm^2^, followed by a decrease, which coincides with the appearance of the O charge peak, which increases quasi exponentially from 3 × 10^15^ cm^−3^ up to 1.15 × 10^16^ cm^−3^. The atomic densities are smaller than the ionic ones by a factor of 10^4^ and follow a similar evolution. This means that for certain fluences, even around the threshold fluence, the ablation process is strongly non stoichiometric. For HfO_2_, the fluences that can be appropriate for pulsed laser deposition would be between 1 and 2 J/cm^2^, while for ZrO_2_, between 1.5 and 2.7 J/cm^2^.

**Figure 8:**
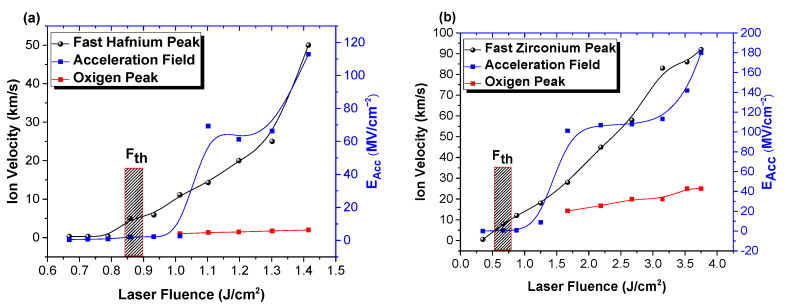
Ion velocities and acceleration field evolution with laser fluence for HfO_2_ (**a**) and ZrO_2_ (**b**).

When analyzing the kinetic energies of the ejected particles, it can be seen from Figure 8 that there is a good correlation between the ion kinetic energy, acceleration field, and overall particle density. The main ionic peaks are selected for both Hf and O. There is a jump of 1 order of magnitude in expansion velocity when the laser fluence is passing the ablation threshold, followed by a quasi-exponential increase with the subsequent increase of laser fluence. Above the ablation threshold, the velocity of Hf ions is found in the 5–48 km/s range, while for the Zr ions in the 8–80 km/s range. The contribution from the positive ions, identified as a contribution from the O ions, defines kinetic ranges between 1 and 2 km/s for the HfO_2_ case and 14 and 25 km/s for the ZrO_2_ plasma. These differences are understandable as there is a factor of 2 between the fluence for which the oxygen contribution can be seen, and thus, the resulting accelerating fields are larger in the case of ZrO_2_. The same difference can be found when looking at the dielectric constant of each irradiated material. This means that the electrical breakdown of the sample can be correlated with the ion acceleration field enhancement during the ablation process. The acceleration field (E_Acc_) [39] is usually generated in the first ps of laser–matter interaction and is a result of charge particle separation. The evolution of the acceleration field with the laser fluence defines a step-like function. For both investigated cases, the E_Acc_ has a jump of 1 order of magnitude at 1 J/cm^2^ for HfO_2_ and 1.6 J/cm^2^ for ZrO_2_, respectively, which coincides with the appearance of the oxygen peak. E_Acc_ is seen increasing from 0.2 MV/cm^2^ characterizing below threshold interactions up to 60–65 MV/cm^2^ above the threshold and continuously increases up to 110 MV/cm^2^. The ZrO_2_ plasma follows a similar evolution, with characteristic values of 0.6 MV/cm^2^, 100 MV/cm^2^, and 120–180 MV/cm^2^ for the below, above threshold and at high irradiation conditions.

To better understand the evolution of the acceleration field and its implication regarding the fundamental ablation mechanism involved in the ejection of the metal and oxygen particles from the targets, simulations were performed in the COMSOL environment. The simulations involved the solving of the heat diffusion equation in three dimensions as well as a function of time. The laser source was defined as a top hat function with a duration of 25 ns, while the targets were modeled as cylinders with 2.54 cm diameter and 0.5 cm thickness. During the simulations, the properties of the materials are considered to be constant and the phase change is not modeled. The top surface of the target model is the active surface, with all of the other boundaries assumed thermally insulated. The heat equation takes into account the absorption and reflection coefficients, the laser power and the spatial distribution of the beam at the impact point, and the depth absorption of the radiation [40]. This represents a first approximation of the heating process and does not take into account the phase change or the consequences to the target physical properties as it acts as a bridge to correlate the E_acc_ to the specific key points during particle removal via thermal ablation mechanism (evaporation, melting, boiling).

**Figure 9:**
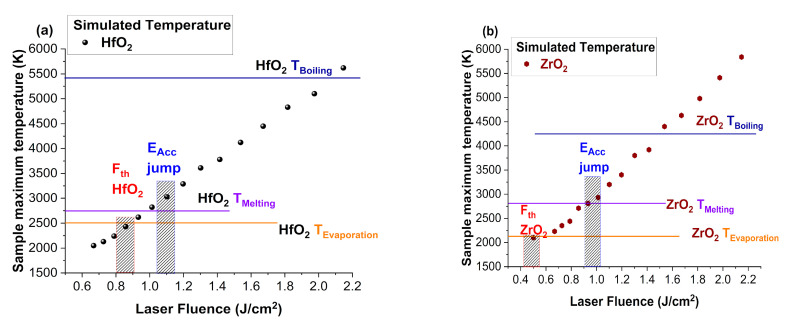
Peak temperature derived from heat diffusion equation for various irradiation conditions of HfO_2_ (**a**) and ZrO_2_ (**b**).

The target temperatures during each irradiation condition were computed and represented in Figure 9. It can be seen that below the threshold fluence the surface temperature is below the evaporation temperature (HfO_2_, ZrO_2_) [41] for both investigated materials. This confirms our conclusions that the measured signal for subthreshold measurements is given by physisorbed and weakly bonded ions on the surface. The subsequent increase of the fluences sees the surface reach and surpass the evaporation temperature, which coincides with the ablation threshold for the two oxides. The lower vapor pressure is expected as the experiments were performed under vacuum conditions. This underlines a possible sublimation of the target before reaching the melting or boiling; thus, in our irradiation conditions, the thermal mechanism occurs even below the threshold and competes with the electrostatic one. The jump in the acceleration field has a resonance in the thermal effects at the target’s surface as it occurs when the melting temperature is reached. Thus, the existence of a molten layer during the ablation process actually enhances the acceleration field, and subsequently, the kinetic energies of the ablated particles. A further increase of the laser fluence creates an environment where explosive boiling can occur, as, even for values below boiling temperature, local defects can promote the formation of vapor bubbles in the melted material and clusters or larger structures can be ejected from the target [42].

## 4. Conclusions

The ablation threshold upon UV ns laser irradiation of HfO_2_ and ZrO_2_ was determined by the unbiased Langmuir probe method. For subthreshold irradiation conditions, the charge current temporal traces follow a classical Maxwell–Boltzmann distribution and corresponds to the ejection of weakly bonded ions from the surface. The LP temporal trace is characterized by the presence of a Coulomb generated peak, attributed to ions with kinetic energies of a few eV. The enhancement of laser fluence leads to the generation of a stronger energetic group of ions with hundreds of eV up to a few keV. Three ablation regimes were identified and correlated with specific features on the LP current. The appearance of positive charges in the plasma leads to an increase in the acceleration field of about one order of magnitude, and thus explains the presence of the high kinetic energy ionic group. The COMSOL simulations of the heat diffusion equation were performed to determine the surface temperature reached for a wide range of irradiation conditions. The ablation threshold fluence corresponds to the surface reaching the evaporation temperature, while the jump in acceleration field and the appearance of the oxygen peak is reflected by reaching the melting point of the material. A strong correlation between the thermal properties of the target material and the behavior of the ablated particles were shown. The data suggest that the thermal mechanisms are dominating the particle removal, even for subthreshold ablation conditions, while electrostatic mechanisms are hindered due to the longer long pulse duration.

## Data Availability

Data will be made available upon request from the corresponding author.
